# Differing conceptual maps of skills for implementing evidence-based interventions held by community-based organization practitioners and academics: A multidimensional scaling comparison

**DOI:** 10.1093/tbm/ibae051

**Published:** 2024-11-20

**Authors:** Shoba Ramanadhan, Jennifer L Cruz, Maggie Weese, Shinelle Kirk, Madison K Rivard, Arthur Eisenkraft, Karen Peterson, Judi Kirk, Albert Whitaker, Chinyere Nwamuo, Scott R Rosas

**Affiliations:** Department of Social and Behavioral Sciences, Harvard TH Chan School of Public Health, Boston, MA 02115, USA; Department of Social and Behavioral Sciences, Harvard TH Chan School of Public Health, Boston, MA 02115, USA; Department of Social and Behavioral Sciences, Harvard TH Chan School of Public Health, Boston, MA 02115, USA; Conservation Law Foundation, Boston, MA 02110, USA; Rhode Island Department of Health, Providence, RI 02908, USA; Curriculum & Instruction, University of Massachusetts Boston, Boston, MA 02125, USA; Community Benefits, Tufts Medicine, Burlington, MA 01803, USA; Boys and Girls Club of Worcester, Worcester, MA 01610, USA; American Heart Association, Waltham, MA 02451, USA; St. Mark Congregational Church, Boston, MA 02121, USA; Department of Social and Behavioral Sciences, Harvard TH Chan School of Public Health, Boston, MA 02115, USA; Concept Systems, Inc., Ithaca, NY, USA; Public Health and Preventive Medicine, SUNY Upstate Medical University, Syracuse, NY, USA

**Keywords:** evidence-based interventions, implementation science, community-based organizations, capacity-building, group concept mapping, health inequities

## Abstract

Community-based organizations (CBOs) are critical for delivering evidence-based interventions (EBIs) to address cancer inequities. However, a lack of consensus on the core skills needed for this work often hinders capacity-building strategies to support EBI implementation. The disconnect is partly due to differing views of EBIs and related skills held by those typically receiving versus developing capacity-building interventions (here, practitioners and academics, respectively). Our team of implementation scientists and practice-based advisors used group concept mapping to engage 34 CBO practitioners and 30 academics with experience addressing cervical cancer inequities implementing EBIs. We created group-specific maps of skills using multidimensional scaling and hierarchical cluster analysis, then compared them using Procrustes comparison permutations. The 98 skills were sorted into six clusters by CBO practitioners and five by academics. The groups generated maps with statistically comparable underlying structures but also statistically significant divergence. Some skill clusters had high concordance across the two maps, e.g. “managing funding and external resources.” Other skill clusters, e.g. “adapting EBIs” from the CBO practitioner map and “selecting and adapting EBIs” from the academic map, did not overlap as much. Across groups, key clusters of skills included connecting with community members, understanding the selected EBI and community context, adapting EBIs, building diverse and equitable partnerships, using data and evaluation, and managing funding and external resources. There is a significant opportunity to combine CBO practitioners’ systems/community frames with the EBI-focused frame of academics to promote EBI utilization and address cancer and other health inequities.

## Teaser

This group concept mapping study draws on the expertise of 34 community-based organizations (CBO) practitioners and 30 academics addressing cervical cancer inequities to explore important differences in conceptualizing core skills needed to implement evidence-based interventions. Convergence and divergence between group-specific skills maps suggest that capacity-building interventions must integrate differing perspectives held by practitioners and academics.

**Implications**

**Practice:** Capacity-building interventions need to be designed to effectively integrate diverse perspectives held by community-based practitioners and academics regarding the core skills required to implement evidence-based interventions.

**Policy:** Integrating the diverse perspectives of community-based practitioners and academics can help policymakers move beyond focusing on making a given evidence-based intervention “work” within harmful systems to initiating action to reshape these systems to center equity.

**Research:** These findings highlight likely gaps related to tailoring capacity-building implementation strategies for practitioners tackling health inequities.

## Background

Evidence-based interventions (EBIs) could prevent about half of the cancers occurring in the USA today, but insufficient and inequitable delivery restricts this potential [1]. Community-based organizations (CBOs) can fill some of the gaps (particularly related to equity), as they have extensive reach and connections within communities impacted by structural discrimination [2]. However, EBI utilization is not the norm in CBOs due to various factors, including time and resource constraints and insufficient training, skills, and support to implement EBIs [3–7]. Building CBO practitioners’ capacity (or skills, knowledge, motivation, resources, and awareness) to find and implement EBIs can increase the impact of these critical interventions [8]. Despite some successful examples, such as the Getting to Outcomes projects [9–11], there is limited research on EBI capacity building in the CBO setting [12, 13]. This is a critical gap, as capacity-building strategies must account for the unique organizational profile of CBOs [3]. They are often understaffed, and personnel may not have had access to public health training. As a result, existing capacity-building approaches may not be suitable (e.g. programs designed for health departments with staff epidemiologists) [3, 14–16]. Additionally, the success of these capacity-building implementation strategies is hindered by a lack of consensus on the skills needed to implement EBIs effectively [3, 5]. Addressing these disconnects is critical, given that there are established access and quality gaps for preventive EBIs among marginalized communities [17].

CBO practitioners and academics appear to conceptualize opportunities to build capacity for implementing EBIs in community settings in meaningfully different ways [18, 19]. The implementation science literature typically identifies a core set of skills for EBI use: assessing context/needs; engaging partners; and selecting, adapting, integrating, evaluating, and sustaining EBIs [8, 20, 21]. Through a series of interviews, we found that CBO practitioners and academics use different frames when thinking about skills for implementing EBIs. Practitioners engaged with EBIs with a long-term health promotion lens, emphasizing individual and community needs. In contrast, academics focused more on the EBI of interest and the broader system connected to that EBI [22]. The differences in their perspectives are critical as academics tend to lead the design and execution of capacity-building interventions. If the offerings are perceived as too narrow for or disconnected from CBO practitioners’ concerns, this can lead to a sense that the training and related EBI may not be relevant for the CBO practitioners and their organizations [19].

To understand conceptualizations of EBI skills in greater depth and refine how these essential implementation strategies are designed, we conducted a group concept mapping (GCM) comparison study focused on expertise held by CBO practitioners and academics working in cancer prevention and health equity. GCM is a multistage, mixed-methods approach that integrates qualitative group processes, such as collaborative brainstorming, with multivariate statistical analyses to generate a shared framework for complex social phenomena [23–25]. This method is commonly used in healthcare and public health and supports the integration of diverse forms of expertise. The approach is becoming increasingly popular within the field of implementation science as well. For example, GCM supported the development of the Expert Recommendations for Implementing Change typology of implementation strategies, the identification of opportunities for alignment between the fields of implementation science and user-centered design, and the conceptualization of barriers to implementation among diverse mental health professionals [26–28].

With the current study, we extended our initial GCM study that integrated data from CBO practitioners and academics to yield a common conceptual map of skills for EBI use. That list of skills covered building diverse and equitable partnerships, connecting with community members, selecting and adapting EBIs, using data and evaluation, and managing implementation [29]. However, formative research for this project [22], combined with insights from practitioners during the process, suggested that a single conceptual map may mask significant differences in perspectives held by practitioners and academics. Thus, we extended our inquiry by assessing whether CBO practitioners and academics utilized meaningfully different conceptual models when considering EBI skills to address cancer inequities. As detailed below, we identified critical areas of overlap and meaningful differences, suggesting a need to align capacity-building targets among practitioners and academics if we are to achieve efficient and equitable EBI delivery.

## Methods

### Data collection and participants

From June to August 2021, we utilized the groupwisdom^TM^ online platform [30] to collect ideas from participants regarding the skills necessary to implement EBIs. Using the web-based platform and asynchronous data collection procedures supported geographic inclusivity and allowed participants to share data anonymously and at their convenience. As is typical of GCM studies, we used a purposive sampling approach to gather diverse perspectives [23]. We recruited from networks held by the project team, project advisors, community partners, state cancer coalitions, the National Cancer Institute Cancer Consortium for Implementation Science, the University of Massachusetts Dana-Farber/Harvard Cancer Center Partnership, and participant nominations. We recruited individuals whose work addressed cervical cancer among marginalized populations. Cervical cancer was a suitable focal health issue for two reasons: (i) there is a rich prevention evidence base (e.g. routine screening), but there are significant inequities between social groups in the USA for disease burden [31–33] and (ii) CBOs are core to strategies increasing community demand and access [34], but the gap in capacity is an important limiting factor. CBO practitioners were eligible if they had worked in public health for at least 5 years and implemented at least one evidence-based cervical cancer prevention program. Academic researchers were eligible if they had 5 or more years of experience designing, implementing, or evaluating evidence-based cancer prevention programs, including at least one focused on cervical cancer.

### GCM methodology

**Figure 1:**
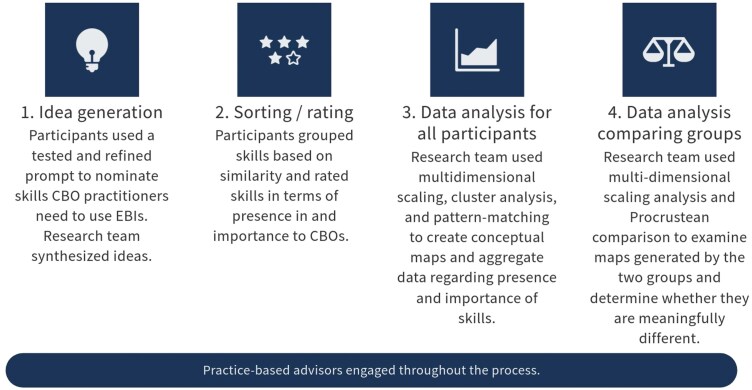
Overview of the group concept mapping process

GCM engages participants over multiple points in time and integrates qualitative data generated through group processes with multivariate statistical analyses to create a shared framework for complex social phenomena [23–25]. The maps that result from this process can then be used as prompts for discussion, integration of diverse expertise, and alignment of action [23, 24]. We built on formative work that explored the range of skills CBO practitioners need to implement EBIs (using cervical cancer EBIs as an example) [22]. In this study (as summarized in Fig. 1), participants were prompted to consider their equity-focused work, emphasizing cervical cancer work. They were then asked to list responses to the following focus prompt: “To use evidence-based interventions, one thing staff of community-based organizations need to know/know how to do is …”

The skills from the idea generation phase were combined with those from the formative research to produce a comprehensive list. The study team pared this list down by collapsing redundant items and removing those considered out of scope. Participants sorted the pared-down list of skills into groups and then offered ratings of the importance and availability of those skills in CBO settings. Details and the map representing a shared model of EBI skills (Steps 1–3) have been reported elsewhere [29].

### Map generation and specification of matrices

Given that our previous research suggested a map combining views of CBO practitioners and academics may mask significant differences, we analysed group-specific data (Step 4 of Fig. 1). For each group, sort data from each participant was compiled into a matrix based on the number of unique skills generated by participants, here a 98 × 98 similarity matrix. Cell values were equal to the number of times skills were sorted together; these values were used as input for the computation of separate concept maps. Thus, one 98 × 98 similarity matrix with values ranging between 0 and 34 was compiled for the CBO practitioners, and another 98 × 98 similarity matrix with values between 0 and 30 was compiled for the academics.

We conducted two-dimensional multidimensional scaling (MDS) analyses of each group’s aggregated, unstructured sort data to create two distinct 98-item point maps [35]. An initial examination of the 98 points arrayed in two-dimensional space provided an overall sense of the location of specific points and the relative distance between each. Skills more closely conceptually related were closer together in space, whereas skills seen as conceptually distinct were farther apart.

After creating the separate point maps, we applied agglomerative hierarchical cluster analysis using the *x*–*y* coordinates as input to partition the space into non-overlapping clusters of points, based on Ward’s algorithm, that presumably reflects meaningful concepts. Three team members (including the lead author) engaged in separate map interpretation and labeling processes for each group. For each group, the core analysts reviewed the results, determined the most appropriate cluster solution (i.e. the number of clusters) for the map, and cooperatively finalized the labeling for the clusters that best described the content within. At that point, the team consulted with the three practice-based advisors to gather their input on the cluster solutions and naming.

### Assessment of map quality and comparability

We examined the respective stress values to assess the internal representational validity of the point maps generated for the two groups. As a goodness-of-fit indicator, stress is measured on a 0–1 scale. A lower stress value indicates better congruence of the map with the raw data. We also computed the correlation between the similarity and distance matrices for the separate maps. Consistent with other multi-map comparison studies, we examined these indicators relative to published meta-analyses (see [36]) to assess the quality and comparability of the group conceptualizations. Finally, we used an adapted version of the Conducting and REporting DElphi Studies reporting checklist [37] to ensure transparent reporting of all study details (Supplementary File 3).

### Procrustes comparison

Following procedures detailed in the literature, a Procrustean comparison approach and permutation strategy were used to examine the extent to which the group-specific two-dimensional point maps resemble each other when matched to maximize fit [25, 38–40]. At that point, the variability of residuals was assessed [41, 42]. Finally, information from the optimal matching of the configurations was generated to examine the concordance of observations for each dimension separately, facilitating the interpretation of similarities and differences between independently produced concept maps.

The first step in the comparison procedure was to use the *x*–*y* coordinates for each point from the group-specific maps as input. Given that the points refer to skills from the same underlying list, a point on one map has a directly corresponding point on the other map. We kept the practitioner map fixed and matched the academic one to it [42]. The Procrustes analysis computed an optimal point-by-point match to minimize the sum of squared deviations and measured the difference between the two maps by aggregating the residual distances between corresponding points. The best-fit solution was identified through residual comparison, with lower residual values indicating points occupy relatively similar positions. This analysis yielded a goodness-of-fit statistic, *m*^2^, and we ran 10 000 random permutations to evaluate the statistical significance of the observed *m*^2^ statistic. We set an α of 0.001 to ensure the relative stability of the estimated *P*-value. This enabled our team to evaluate the probability that *m*^2^ exceeded its critical value and determine if the two matrices showed a strong, non-random resemblance. We examined the residual values produced during the Procrustes analyses to identify patterns among the minimized residual sum of squares between the points of each subgroup point map. To graphically evaluate the point maps, we used the results of the Procrustean algorithm to superimpose the point maps and examine the residuals produced in the analysis against the respective point placement.

We analysed the concept mapping data for each subgroup using the groupwisdom^TM^ web platform to generate separate point maps. We performed the distance matrix computations, point map comparisons, and residual analyses using MS Excel 2021 and IBM SPSS (version 27). Finally, we conducted the Procrustes comparison and permutation test using the PROTEST (PROcrustean randomization TEST) program.

### Study team composition

The academic researchers on the team had expertise in implementation science, CBO-based program delivery, health equity, and qualitative and quantitative research. The project was supported by three advisors with expertise in CBO practice, particularly related to EBI delivery and health equity. Throughout the project, we worked with these advisors as we pared down and refined the list of skills after the idea generation phase, reviewed and interpreted the cluster solution, and determined how best to share results with participants and other constituents. One advisor joined later than the others and contributed to the final two feedback activities. All three co-authored this manuscript. Last, we shared a brief with all participants, including preliminary results and an invitation to share interpretations.

## Results

The group-specific map generation and comparison process included data from 64 participants (34 CBO practitioners and 30 academic researchers). As highlighted in Table 1, participants had 17 years of experience on average, with 14 among CBO practitioners and 21 among academic researchers. A larger proportion of CBO practitioners identified as Black or African American, American Indian or Alaska Native, or Other race than the academic researchers. Participants were from the four Census Bureau-designated regions of the USA, with many academic researcher participants based in the South and many CBO practitioners from the Northeast. Participants served a wide range of communities experiencing health inequities.

**Table 1:** Participants in the group concept mapping activities (*n* = 64)

	Academics (*n* = 30)	CBO practitioners (*n* = 34)	Total (*n* = 64)
Region			
South	16	11	27
Northeast	9	14	23
West	3	2	5
Midwest	2	7	9
Populations served^a^			
Low-income	24	24	48
Black or African American	21	22	43
Latinx/Hispanic	19	21	40
Rural	17	15	32
Asian	8	11	19
LGBTQ+	7	15	22
American Indian or Alaska Native	5	8	13
Pacific Islander	2	4	6
Participant race^a^			
White	24	17	41
Black or African American	1	10	11
Asian	4	3	7
American Indian or Alaska Native	0	2	2
Other race	1	2	3
Prefer not to say	0	2	2
Ethnicity			
Hispanic/Latinx	3	3	6
Years of research and/or practice experience (mean, standard deviation)	20.83 (10.14)	13.78 (8.31)	17.14 (9.81)

^a^Multiple selections permitted.

### Map construction and specification of matrices

**Figure 2:**
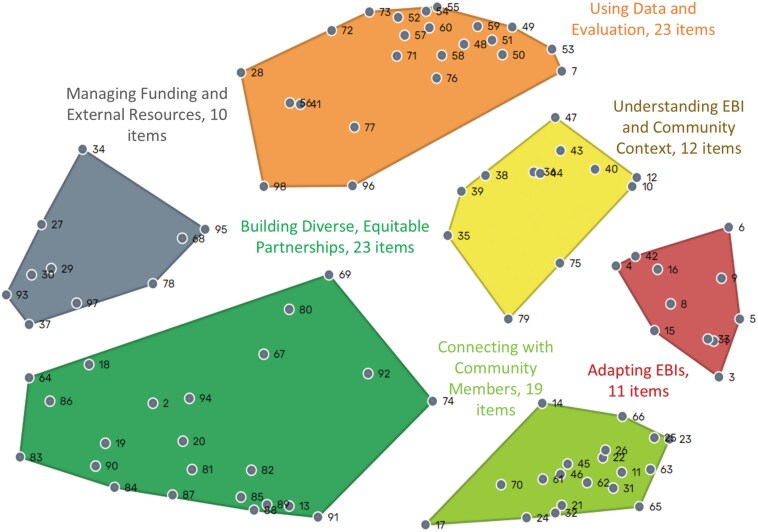
Conceptual map developed by CBO practitioners (*n* = 34 participants, 98 skills). EBI, evidence-based intervention

**Figure 3:**
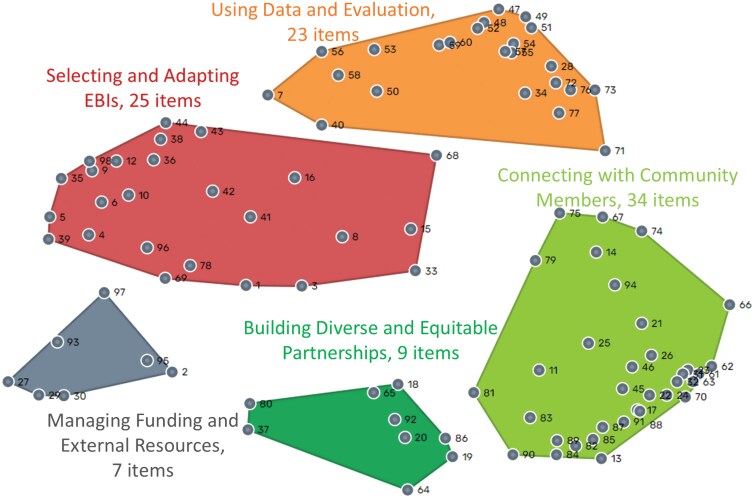
Conceptual map developed by academics (*n* = 30 participants, 98 skills). EBI, evidence-based intervention

Separate MDS analyses of the sorting data resulted in two-dimensional maps with stress values of 0.25 after nine iterations for CBO practitioners and 0.23 after 14 iterations for academics. These values fell within the range that indicated concordance between the matrix data and the representations in the maps and were within the range found in previous meta-analytic studies of GCM [36]. The correlation between the similarity and distance matrices for both maps was 0.77 (*P* < .001). Both were at the high end of the range found in studies of similarly constructed point maps [36] and did not differ significantly from each other (*z* = 0, *P* = .5). This indicates there was no difference in the magnitude of the relationship between the sorting and distance matrices of the separate maps and thus, it was appropriate to compare the two conceptualizations. The resultant point maps for CBO practitioners and academics are shown in Figs. 2 and 3, respectively. The full list of skills is presented in Supplementary File 1.

The separate clustering processes for the two groups resulted in different cluster solutions based on the location of points plotted on the independently generated maps. The separate point-cluster maps for CBO practitioners and academics are shown in Figs. 2 and 3, respectively. The best cluster solution included six clusters for the map based only on CBO practitioners’ data. As seen in Fig. 2, the clusters included: (i) *Using data and evaluation*, (ii) *Understanding the selected EBI and community context*, (iii) *Adapting EBIs*, (iv) *Connecting with community members*, (v) *Building diverse and equitable partnerships*, and (vi) *Managing funding and external resources*.

For the map based on data from academics only, the best cluster solution included five clusters (Fig. 3), including (i) *Using data and evaluation*, (ii) *Connecting with community members*, (iii) *Building diverse and equitable partnerships*, (iv) *managing funding and external resources*, and (b) *Selecting and adapting EBIs*.

### Procrustes comparison using PROTEST

Our analysis of the spatial correspondence between the two maps produced an *m*^2^ goodness-of-fit statistic. We found *m*^2^ = 0.63; *P* < .0001, meaning the correspondence was greater than expected due to chance (i.e. the two maps showed a pattern of similarities that were not random and possessed a comparable underlying structure). Based on a previous categorization scheme for *m*^2^ values, 0.63 suggests average similarity, although caution in applying the categories is warranted since they are not validated within a sample of GCM studies [43]. These findings suggest that the two maps were more similar than expected due to chance alone, yet there were meaningful levels of dissimilarity.

Supplementary Material shows each numbered point plotted according to their respective residual value for each dimension and displays the distribution of the minimized sum of square results. Points plotted closest to the *x*–*y* axis intersection possessed the lowest residual values along both dimensions (e.g. 75, 53, 30, and 29). In contrast, the points plotted furthest from the *x*–*y* axis intersection possessed the highest residual values (e.g. 5, 34, 6, and 9). Plotting the residuals in this manner allowed us to distinguish points proximally vs. distally located in the superimposed comparison.

**Figure 4:**
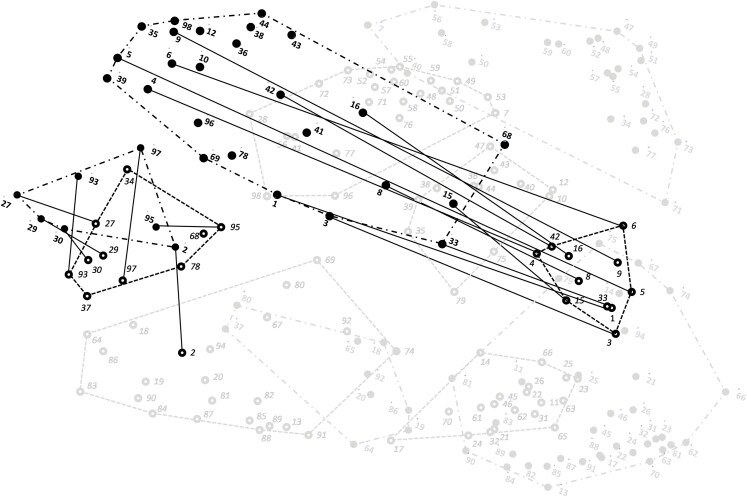
Overlay of group-specific maps (*n* = 64 participants, 98 skills). Practitioners’ data is marked with dashed lines indicating cluster boundaries and academics’ data is marked with dot-dashed lines indicating cluster boundaries. Numbers refer to discrete skills (listed in Supplementary File 1) and black lines connecting paired skills reflect residual differences between locations on the respective maps

To illustrate the differences, the CBO practitioner point-cluster point map was superimposed on and fitted to the academic point-cluster map as the reference target, shown in Fig. 4.

Visually, we examined the cluster content similarities and differences by observing how the points in the CBO practitioner map (organized and marked by cluster with dashed lines) were distributed differently in the academic map (organized and marked by cluster with dash-dot lines). The proximity of points to one another is meaningful in GCM output. Differences in location reflect the discerning judgments of participants about the similarity among items during sorting and ultimately affect the conceptual meaning of the group’s specific representation. An example of the similarities and differences in the overlap of the concepts can be seen in Fig. 4. Of note, on the left-hand side of the map, the overlap between the dark dash-dot bounded cluster (“managing funding and external resources” from the academic map) and the dark dash bounded cluster (“managing funding and external resources” from the practitioner map) was evident. Proximally, the two clusters were in the same general area, and conceptually, nearly all of the same items were closely positioned. The lines drawn between pairs of points were relatively short and corresponded to the lower end of the residual values found in Supplementary Material. This high overlap reflects similar conceptualizations of the construct by CBO practitioners and academics.

Conversely, in the center of the map, the overlap between the sizeable dark dash-dot bounded cluster (“selecting and adapting EBIs” from the academic map) and the dark dash bounded cluster (“adapting EBIs” from the practitioner map) is very different. Proximally, the two clusters did not overlap, and conceptually, nearly all of the same items were positioned relatively far apart. The lines drawn between pairs of points were rather long and corresponded to the high end of the residual values found in Supplementary Material. This low degree of overlap reflects discord between the groups as to the conceptual similarity of the construct based on independent judgments during sorting. Indeed, the tightly packed set of items for the CBO practitioner cluster “adapting EBIs” reflected a coherent conceptual construct. In contrast, the cluster was much more diffuse and broad for the same items on the academic map.

Additionally, as shown in Supplementary File 2, the distribution of skills across clusters in the two maps offered additional insight into how CBO practitioners and academics had overlapping but ultimately different conceptual maps. We further examined the similarities and differences in how CBO practitioners and academics clustered skills by comparing cluster listings from the two groups. We noted by color the overlap in how each respective group organized the skills in similar constructs. Several clusters represented common skill sets, as reflected in the labels and the large number of shared items. Substantial overlap was noted in the “using data and evaluation,” “connecting with community members,” and “managing funding and external resources,” suggesting high cross-group agreement in these areas. In contrast, CBO practitioners emphasized the skills related to adapting EBIs and selecting EBIs based on the community context as two separate constructs, while academics conceptualized that set of skills as a single construct. It should be noted that although there was overlap among the two groups in the “building diverse and equitable partnerships” and “connecting with community members” content, the clustering of the skills was meaningfully different. CBO practitioners placed many of those items in the partnership cluster, whereas academics organized most of the content in those two clusters in “connecting with community members.”

## Discussion

We found both overlaps and statistically meaningful differences between the conceptual models CBO practitioners and academics used regarding EBI skills to address cancer inequities. This suggests that the design and execution of capacity-building implementation strategies will need to actively integrate and align the perspectives of CBO practitioners and academics if they are to have the intended impact. Combining these different but complementary perspectives through effective partnerships to create meaningful and sustainable impact requires a thorough understanding of how these groups conceptualize what is necessary to address cancer inequities.

First, we found that many clusters addressed similar concepts across the two groups and reflected prominent models of EBI use in CBOs, which emphasize finding data, creating partnerships, selecting and adapting EBIs, and evaluating implementation [11, 20]. However, consistent with other work emphasizing practitioners’ perspectives, various practical skills, such as addressing funding and resources, were also added [44, 45]. The explicit focus by CBO practitioners and academics on community engagement as a set of core skills (rather than an approach that encapsulates EBI efforts) differs from many standard measures of capacity for EBI use [46]. Another important finding was the discrete category created by practitioners that focused on understanding the selected EBI and the community context. The implementation context is dramatically understudied despite its prominence in implementation science theories, models, and frameworks [47–50]. There is an opportunity to draw on practice-based expertise to fill this gap.

**Figure 5:**
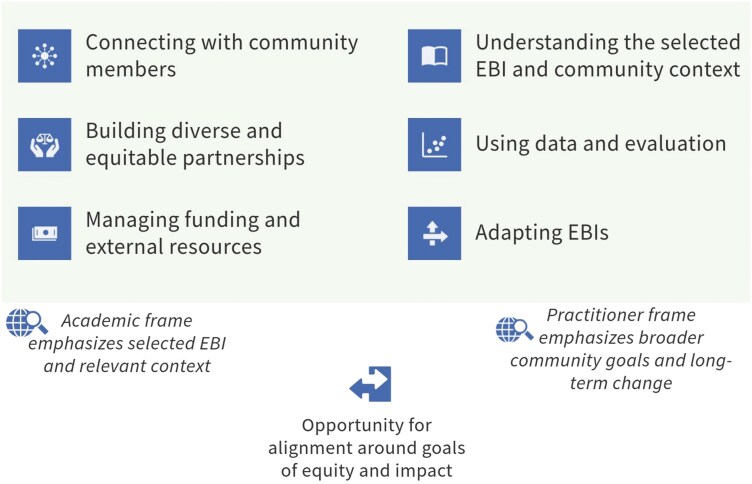
Core skills identified by CBO practitioners and academics for implementing EBIs in CBOs to advance health equity

Second, this exercise confirmed our expectation that the two maps would be more similar than expected by chance but still meaningfully different. As an example of overlap, common clusters of skills included “managing funding and external resources” and “using data and evaluation.” As an example of distinct conceptualization, “building diverse and equitable partnerships” and “connecting with community members” were identified as core skills for both groups, but many of the individual skills that CBO practitioners assigned to “building diverse and equitable partnerships” were assigned to “connecting with community members” by academics. As another example of divergence, many of the skills that CBO practitioners placed in “understanding the selected EBI and community context” and “adapting EBIs” appeared in a single construct for academics—“selecting and adapting EBIs.” We connect these findings to our previous work identifying the broad perspective taken by CBO practitioners (centering the community and long-term health promotion goals) and the more targeted perspective taken by academics (centering the delivery of a given EBI) [22]. Integrating these perspectives in capacity-building interventions is vital to fully leveraging both groups’ expertise. Figure 5 offers a synthesis of core skills that takes the broadest perspective of skills across groups.

An essential set of opportunities to align perspectives and bridge disconnects comes from a “participatory implementation science” approach, which uses collaboration and co-direction to center diverse expertise and supports joint solution creation to achieve community, practice, and scientific goals [51]. Applied to capacity-building, a vital starting point could come from academics engaging CBO practitioners and other public health leaders to develop capacity-building interventions. As prompted by Leeman’s framework [8], such collaboration would need to focus on building the necessary knowledge, skills, motivations, and resources for CBO practitioners to implement EBIs. This work must address the strategies used (e.g. training or technical assistance) and the structure (e.g. delivery methods).

We present these findings in the context of a set of limitations. First, we grounded our inquiry in the example of cervical cancer inequities, which may impact the types of skills respondents nominated and how they sorted them. However, we felt it was essential to provide a concrete example of a health inequity amenable to preventive action in community settings. Second, we cannot make claims about the representativeness of these maps. Instead, we base the strength of the findings on sample sizes that were fairly typical of other GCM exercises [36] and our effective recruitment of a highly experienced set of practitioners and academics who worked with a wide range of populations experiencing cancer inequities across a range of areas in the USA. Finally, we recognize the limitations of restricting participation to those sufficiently comfortable in English to participate in study activities. Given the broad range of populations of interest and language’s key role in the process, it seemed appropriate for an initial inquiry. A significant strength of the study is the equal emphasis on expertise held by practitioners and academics through the concept mapping process and recruitment of similar numbers of experts from each group. The rich literature on participatory research highlights the importance of leveraging varied knowledge and experiences that diverse actors bring to the issue [51]. The study team also included three practice-based advisors, who were able to shape the study to reflect the realities of implementing EBIs in CBOs for health equity work and served as co-authors on the manuscript.

The following steps with this research should examine how different conceptualizations should influence the design and execution of capacity-building implementation strategies. Additionally, while the core list of skills resonates with the literature, it is worth assessing which skills must be present among all CBO practitioners (e.g. connecting with community members) versus among a select subset, such as program managers (e.g. managing funding and external resources). This will allow for the further refinement of vital capacity-building activities.

## Conclusions

In summary, this study allowed us to evaluate the convergence and divergence between how CBO practitioners and academics conceptualize skills for EBI utilization to address cancer inequities. Our findings suggest that capacity-building interventions must be designed to integrate the similarities and differences in the two groups’ conceptualizations to increase the uptake and utilization of EBIs in community settings. Centering the diverse expertise of these groups to allow for both EBI implementation and a broader systems perspective is consistent with our goals to move implementation science efforts beyond a focus on making a given EBI “work” within harmful systems to initiating action to reshape these systems to center equity [51].

## Supplementary data

Supplementary material is available at *Translational Behavioral Medicine* online.

ibae051_suppl_Supplementary_File_3

ibae051_suppl_Supplementary_File_1

ibae051_suppl_Supplementary_File_2

## Data Availability

The datasets used and/or analysed during the current study are available from the corresponding author upon reasonable request.

## Welfare of Animals

This article does not contain any studies with animals performed by any of the authors.
