# Supplementary material for: Differing conceptual maps of skills for implementing evidence-based interventions held by community-based organization practitioners and academics: A multidimensional scaling comparison
Source: Transl Behav Med. 2024 Nov 20;15(1):ibae051. doi: 10.1093/tbm/ibae051 (PMC11756311; doi:10.1093/tbm/ibae051)
Supplement: ibae051_suppl_Supplementary_File_3 [file ibae051_suppl_supplementary_file_3.docx]

**Supplemental File 3:** Reporting of Group Concept Mapping (GCM) procedures using adapted CREDES guidelines [22]

| Rationale | Where in the article it is described |
| --- | --- |
| Justification. The choice of the GCM technique as a method of systematically collating expert consultation and building consensus needs to be well justified. When selecting the method to answer a particular research question, it is important to keep in mind its constructivist nature | Intro (p.3)  Methods (p.4) |
| Planning and design |  |
| Planning and process. The GCM technique is a flexible method and can be adjusted to the respective research aims and purposes. Any modifications should be justified by a rationale and be applied systematically and rigorously | n/a |
| Study conduct |  |
| Informational input. All material provided to the expert panel at the outset of the project and throughout the Delphi process should be carefully reviewed and piloted in advance in order to examine the effect on experts’ judgements and to prevent bias | The materials focused on highlighting the diverse expertise held by CBO practitioners and academics. |
| Prevention of bias. Researchers need to take measures to avoid directly or indirectly influencing the experts’ judgements. If one or more members of the research team have a conflict of interest, entrusting an independent researcher with the main coordination of the study is advisable | The web-based platform and ability to engage in brainstorming anonymously prevented researcher influence. |
| Interpretation and processing of results. Consensus does not necessarily imply the ‘correct’ answer or judgement; (non)consensus and stable disagreement provide informative insights and highlight differences in perspectives concerning the topic in question | We used standard qualitative procedures to identify areas of convergence, while also seeking out and attending to divergent cases |
| External validation. It is recommended to have the final draft of the resulting guidance reviewed and approved by an external board or authority before publication and dissemination | Three practice-based advisors were engaged throughout the process. Methods (p.8) |
| Reporting |  |
| Purpose and rationale. The purpose of the study should be clearly defined and demonstrate the appropriateness of the use of the GCM technique as a method to achieve the research aim. | Introduction (p.3) |
| Expert panel. Criteria for the selection of experts and transparent information on recruitment of the expert panel, socio-demographic details including information on expertise regarding the topic in question, (non)response and response rates over the ongoing iterations should be reported | Methods (p.4) for selection  Results (p.8) for participant details |
| Description of the methods. The methods employed need to be comprehensible; this includes information on preparatory steps (How was available evidence on the topic in question synthesised?), piloting of material and survey instruments, design of the survey instrument(s), the number and design of survey rounds, methods of data analysis, processing and synthesis of experts’ responses to inform the subsequent survey round and methodological decisions taken by the research team throughout the process | These methods are described in detail in a previous paper, cited in the Methods (p.4) |
| Procedure. Flow chart to illustrate the stages of the process, including a preparatory phase, data collection, interim steps of data processing and analysis, and concluding steps | Methods (p.5) |
| Definition and attainment of consensus. It needs to be comprehensible to the reader how consensus was achieved throughout the process, including strategies to deal with non-consensus | n/a |
| Results. Reporting of results for each step | Results for steps 1-3 are reported in a previous paper, cited in the Methods (p.4). Results for each relevant step for this analysis are presented in sequential order in the Results (starting p.8) |
| Discussion of limitations. Reporting should include a critical reflection of potential limitations and their impact of the resulting findings | Discussion (p.18) |
| Adequacy of conclusions. The conclusions should adequately reflect the outcomes of the Delphi study with a view to the scope and applicability of the resulting findings | Discussion (p.18) |
