# Supplementary material for: Differing conceptual maps of skills for implementing evidence-based interventions held by community-based organization practitioners and academics: A multidimensional scaling comparison
Source: Transl Behav Med. 2024 Nov 20;15(1):ibae051. doi: 10.1093/tbm/ibae051 (PMC11756311; doi:10.1093/tbm/ibae051)
Supplement: ibae051_suppl_Supplementary_File_2 [file ibae051_suppl_supplementary_file_2.docx]

Supplemental File 2. Distribution of skills across clusters for practitioner and academic maps.

| Practitioners | | |  | Academics | | |
| --- | --- | --- | --- | --- | --- | --- |
| Cluster # | Cluster Label | Skills |  | Cluster # | Cluster Label | Skills |
| 1 | Adapting EBIs | **1, 3, 4, 5, 6, 8, 9, 15, 16, 33, 42** |  | 1 | Selecting and Adapting EBIs | **1, 3, 4, 5, 6, 8, 9, 10, 12, 15, 16, 33, 35, 36, 38, 39, 41, 42, 43, 44, 68, 69, 78, 96, 98** |
| 2 | Managing Funding and External Resources | **27, 29, 30, 34, 37, 68, 78, 93, 95, 97** |  | 2 | Managing Funding and External Resources | **2, 27, 29, 30, 93, 95, 97** |
| 3 | Using Data and Evaluation | **7, 28, 41, 48, 49, 50, 51, 52, 53, 54, 55, 56, 57, 58, 59, 60, 71, 72, 73, 76, 77, 96, 98** |  | 3 | Using Data and Evaluation | **7, 28, 34, 40, 47, 48, 49, 50, 51, 52, 53, 54, 55, 56, 57, 58, 59, 60, 71, 72, 73, 76, 77** |
| 4 | Building Diverse and Equitable Partnerships | **2, 13, 18, 19, 20, 64, 67, 69, 74, 80, 81, 82, 83, 84, 85, 86, 87, 88, 89, 90, 91, 92, 94** |  | 4 | Building Diverse and Equitable Partnerships | **18, 19, 20, 37, 64, 65, 80, 86, 92** |
| 5 | Connecting with Community Members | **11, 14, 17, 21, 22, 23, 24, 25, 26, 31, 32, 45, 46, 61, 62, 63, 65, 66, 70** |  | 5 | Connecting with Community Members | **11, 13, 14, 17, 21, 22, 23, 24, 25, 26, 31, 32, 45, 46, 61, 62, 63, 66, 67, 70, 74, 75, 79, 81, 82, 83, 84, 85, 87, 88, 89, 90, 91, 94** |
| 6 | Understanding the Selected EBI and Community Context | **10, 12, 35,** **38, 39, 40, 43, 44, 47, 75, 79** |  |  |  |  |
